# Predicting the Population Risk of Suicide Using Routinely Collected Health Administrative Data in Quebec, Canada: Model-Based Synthetic Estimation Study

**DOI:** 10.2196/52773

**Published:** 2024-06-28

**Authors:** JianLi Wang, Fatemeh Gholi Zadeh Kharrat, Geneviève Gariépy, Christian Gagné, Jean-François Pelletier, Victoria Kubuta Massamba, Pascale Lévesque, Mada Mohammed, Alain Lesage

**Affiliations:** 1 Department of Community Health and Epidemiology Faculty of Medicine Dalhousie University Halifax, NS Canada; 2 Institut intelligence et données Université Laval Quebec City, QC Canada; 3 Centre for Surveillance and Applied Research Health Promotion and Chronic Disease Prevention Branch Public Health Agency of Canada Ottawa, ON Canada; 4 Department of Psychiatry University of Montreal Montreal, QC Canada; 5 Institut national de santé publique du Québec Quebec City, QC Canada

**Keywords:** population risk prediction, case-control, development, validation, health administrative data, suicide, depression, anxiety, Quebec, Canada, mental health, suicide prevention, prevention, adolescent, adolescents, teen, teens, teenager, teenagers, male, female

## Abstract

**Background:**

Suicide is a significant public health issue. Many risk prediction tools have been developed to estimate an individual’s risk of suicide. Risk prediction models can go beyond individual risk assessment; one important application of risk prediction models is population health planning. Suicide is a result of the interaction among the risk and protective factors at the individual, health care system, and community levels. Thus, policy and decision makers can play an important role in suicide prevention. However, few prediction models for the population risk of suicide have been developed.

**Objective:**

This study aims to develop and validate prediction models for the population risk of suicide using health administrative data, considering individual-, health system–, and community-level predictors.

**Methods:**

We used a case-control study design to develop sex-specific risk prediction models for suicide, using the health administrative data in Quebec, Canada. The training data included all suicide cases (n=8899) that occurred from January 1, 2002, to December 31, 2010. The control group was a 1% random sample of living individuals in each year between January 1, 2002, and December 31, 2010 (n=645,590). Logistic regression was used to develop the prediction models based on individual-, health care system–, and community-level predictors. The developed model was converted into synthetic estimation models, which concerted the individual-level predictors into community-level predictors. The synthetic estimation models were directly applied to the validation data from January 1, 2011, to December 31, 2019. We assessed the performance of the synthetic estimation models with four indicators: the agreement between predicted and observed proportions of suicide, mean average error, root mean square error, and the proportion of correctly identified high-risk regions.

**Results:**

The sex-specific models based on individual data had good discrimination (male model: C=0.79; female model: C=0.85) and calibration (Brier score for male model 0.01; Brier score for female model 0.005). With the regression-based synthetic models applied in the validation data, the absolute differences between the synthetic risk estimates and observed suicide risk ranged from 0% to 0.001%. The root mean square errors were under 0.2. The synthetic estimation model for males correctly predicted 4 of 5 high-risk regions in 8 years, and the model for females correctly predicted 4 of 5 high-risk regions in 5 years.

**Conclusions:**

Using linked health administrative databases, this study demonstrated the feasibility and the validity of developing prediction models for the population risk of suicide, incorporating individual-, health system–, and community-level variables. Synthetic estimation models built on routinely collected health administrative data can accurately predict the population risk of suicide. This effort can be enhanced by timely access to other critical information at the population level.

## Introduction

Each year, over 4500 Canadians take their own life [1], and more than 700,000 people die because of suicide worldwide [2]. As such, suicide has become a major international public health challenge. To facilitate suicide prevention, mechanisms should be in place that enable policy and decision makers to make informed decisions and mobilize resources to high-risk populations at the right places before tragic events occur. To achieve this goal, methods of predicting the population risk of suicide are critical.

Many suicide risk assessment tools have been developed in clinical settings with the hope that identifying patients at high risk and providing needed mental health services would reduce an individual’s risk of suicide [3,4]. Going beyond individual risk assessment, one important application of risk prediction models is population health planning [4]. In the realm of population health, population refers to “a group of individuals, in contrast to the individuals themselves, organized into many different units of analysis, depending on the research or policy purpose” [5]. These different units may be geographic regions (eg, states, provinces, and cities), groups (eg, workplaces and schools), and policy-relevant subpopulations (eg, by sex/gender and ethnicity). The aggregate health of the populations in these units is the focus of policy and decision makers through population health planning and policy [5]. Suicide has a complex etiology and is a result of the interaction of the risk and protective factors at the individual, health care system, and community levels [6-13]. Therefore, policy and decision makers and mental health service planners can play an important role in suicide prevention. However, policy and decision makers need tools that allow them to identify communities/regions that are at high risk so that they can mobilize resources and complex population-based interventions to these high-risk regions in advance. Ideally, such tools are developed based on readily accessible and real-time data so that they can closely monitor the population risk and the effects of the interventions.

There is a paucity of prediction models for the population risk of suicide. Gradus and colleagues [14] developed sex-specific machine learning algorithms for suicide using data from eight Danish national health and social registries. Kessler et al’s [15] machine learning algorithms targeted US Army soldiers who were hospitalized. Predictive models for population risk may use not only individual data but also health system–level (eg, quality of mental health care and mental health budget) and community-level data (eg, unemployment rate and social deprivation levels in the community). Therefore, a limitation of these individual-oriented models is the lack of consideration of health care system– and community-level factors as well as the potential changes in the distributions of the predictors over time. Research [16-18] has shown that community-level social vulnerability and antidepressant use/prescription are significantly associated with suicide at the population level. Kandula et al [19] went further by building a model to predict county-level suicide mortality in the United States using county-level annual measures of socioeconomic predictors of suicide risk and state-level prevalence of major depressive episodes and firearm ownership. Kandula et al’s [19] model used data from a variety of US public data sources, which makes the application of the model highly feasible. However, health administrative data was not one of the data sources. Studies from different regions have shown that a large proportion of people contacted health services in the year before their death [20-23]. For example, Canadian studies found that 82% of suicide decedents contacted health services in the year before their death [24] and that over 50% specifically contacted mental health services [25]. Therefore, the role of health service use data in predicting suicide risk at the population level cannot be ignored. The objective of this study was to develop and validate sex-specific predictive models for the population risk of suicide based on individual-, health system–, and community-level indicators in Quebec, Canada.

## Methods

### Population and Setting

The target population is the general population aged ≥15 years residing in the province of Quebec, Canada. Health care in Canada is provided through provincial and territorial systems of publicly funded health care that are universally accessible. In Quebec, health services are planned and delivered through 18 health regions, 22 integrated health and social services centers, and 166 local community services centers. Budgetary decisions are made at the levels of province and health regions.

### Data Sources

We linked 5 health administrative databases using residents’ health care insurance numbers and the Canadian Urban Environmental Health Research (CANUE) data by postal codes, including the vital statistics death database, the physician claims database, the hospital discharge database, the Insured Person Registration File, and the public drug plan. The data of these databases (eg, billing and service procedures codes and service dates) are routinely submitted by clinics and hospitals for billing and administration purposes. No self-reported data were collected from residents. These databases cover up to 98% of the population in Quebec. Together, the 5 health administrative databases can provide information on individual- (eg, sex and age), program- (eg, hospitalization and emergency department visits), and system-level (eg, mental health and addiction budgets) indicators [8]. CANUE is a Canadian consortium database [26], which contains indicators for social deprivation, material deprivation, and built environment at the community level. The data linkage was performed at the Quebec Institute of Public Health (INSPQ) where the health administrative databases and CANUE data are kept. This study was approved by the Health Sciences Research Ethics Board of Dalhousie University (2021-5913). The methodological details of this study can be found in a previous publication [27].

### Study Design

Because the base rate of suicide in the population is low, we used a case-control study design to develop sex-specific suicide risk predictive algorithms. For the training data set, we included all death by suicide cases that occurred from January 1, 2002, to December 31, 2010. The control group was a 1% random sample of living individuals in each year between January 1, 2002, and December 31, 2010, from the Quebec physician claim database. Individuals in the control group were only allowed to be selected once. The cases and controls were not matched to allow for maximum variability in predictors. The health administrative data from January 1, 2011, to December 31, 2019, were used for validation.

### Suicide

Death by suicide cases were ascertained by Quebec’s Coroner’s Office after investigation. The decision is registered in the Quebec vital statistics database.

### Predictors

Individual, programmatic, systemic, and community factors (Multimedia Appendix 1) 5 years before the suicide event, or the index date for controls, were used as candidate predictors to develop the risk predictive algorithms. For example, we extracted the data about the diagnosis of major depression (an individual-level factor) in the past 6, 12, 24, 48, and 60 months as 5 separate candidate predictors. Similarly, we extracted the annual mental health and addiction budget of each health region (a systemic-level factor) in the past 5 years as candidate predictors. The percentages of missing values associated with the variables in the databases ranged from 0.87% to 4.12%. The initial selection of candidate predictors was determined by content knowledge (ie, known relationships between suicide or suicide behaviors and individual- and local area–level variables), clinical utility, and policy relevance through team meetings (Multimedia Appendix 1).

### Model Development

We included all preselected variables in penalized least absolute shrinkage and selection operator (LASSO) regression. The LASSO penalization factor selects important predictors by shrinking coefficients for weaker predictors toward zero, excluding predictors with estimated zero coefficients from the final sparse prediction model. We performed a correlation analysis among variables selected by LASSO regression and identified variables that were strongly correlated (γ≥0.60). Correlated variables were discussed by team members, and the variables that have better policy implication and clinical utility were kept and became the candidate predictors for model development.

We used logistic regression to develop the sex-specific statistical models. The backward selection method was used to eliminate unpredictive variables and to identify the model with the best calibration and discrimination. The decisions of model selection were initially based on the changes in the values of the Akaike information criterion and Bayesian information criterion [28]. Prediction accuracy was assessed by the discrimination and calibration of the model. Discrimination is the ability of a prediction model to separate those who experienced the outcome events from those who did not. We quantified this by calculating the C statistic, analogous to the area under a receiver operating characteristic curve. C statistic ranges from 0.5 to 1, with a higher value indicating better discrimination. A C statistic of 0.7, 0.8, and 0.9 may be considered the threshold for acceptable, good, and excellent discrimination, respectively. Calibration measures how closely predicted outcomes agree with actual outcomes. We used the Brier score to measure calibration. Brier score is the mean squared difference between the predicted probability and the actual outcome. The lower the Brier score is for a set of predictions, the better the predictions are calibrated. Given that the program will be used to forecast population risk by policy and decision makers, we prioritized calibration over discrimination in model development.

The second step of model development was to estimate the synthetic proportions of suicide. A synthetic estimate is a prevalence estimate for a local area that is calculated by using descriptive or demographic data at the community level [29]. The model-based synthetic estimation consists of two stages. First, for each predictor, the proportion of individuals within each category of that predictor in the initial modeling was computed separately by region. For instance, if hospitalization due to a suicide attempt in the past 5 years is a predictor in the model, the proportion of individuals with this attribute in a specific health region is calculated. If age is a continuous variable in the model, the mean age of the population in a health region is calculated. As such, the synthetic model contained community characteristics as predictors. A syntax program was then developed to apply the regression coefficients to the corresponding proportions and means in the data set, and to calculate the logit estimates for each of the health regions. The resulting logit values for each of the health regions were then converted into probabilities (ie, the synthetic estimate of the risk of suicide in the health region).

### Validation

The validation data set included all suicide cases and 1% of controls from January 1, 2011, to December 31, 2019. We first calculated the yearly proportion of suicide at the provincial and health regional levels for males and females (ie, observed proportion). We then applied the developed synthetic models to the validation data to estimate the yearly prevalence of suicide death at the provincial and health regional levels in males and females (ie, predicted proportion). We visually compared and calculated the absolute differences between the predicted and observed risks; smaller differences indicate better calibration with the data and model accuracy. Additionally, we assessed the synthetic model performance using three indicators: mean average error (MAE), root mean square error (RMSE), and the proportion of correctly identified high-risk regions. The MAE is the average magnitude of the difference between the predicted and observed suicide death rate for each health region. The RMSE is the square root of the average magnitude of the difference squared and is, therefore, similar to MAE but penalizes prediction errors with greater magnitude. More accurate predictions will result in smaller MAE and RMSE. To assess the extent to which high-risk regions are correctly identified, the top 5 health regions with the highest predicted and observed suicide risks were identified. The proportion of health regions observed in the top quartile of observed suicide death risks that were rightly predicted to be in the top 5 was calculated.

### Ethical Considerations

This study was approved by the Health Sciences Research Ethics Board of Dalhousie University (REB number: 2021-5913), which waived the need for informed consent. This study used existing data held by the INSPQ, which are routinely collected by the provincial government. Under provincial health information regulations, deidentified INSPQ data were used, as requested, in the context of epidemiological surveillance of suicide, with ethical approval. With the deidentified data, individual patients cannot be identified, and the results of this study were reviewed and vetted by the INSPQ before publication. No compensation was provided to individuals in the databases.

## Results

The demographic and socioeconomic characteristics of participants in the training data are in Table 1. Between January 1, 2002, and December 31, 2010, there were 8899 suicide cases (6713 males and 2186 females). We included a 1% random sample of the Quebec general population as controls (316,574 males and 329,016 females). Most of the participants lived in urban areas. Participants were grouped by quartile values of social deprivation and material deprivation scores based on population norms.

**Figure 1:**
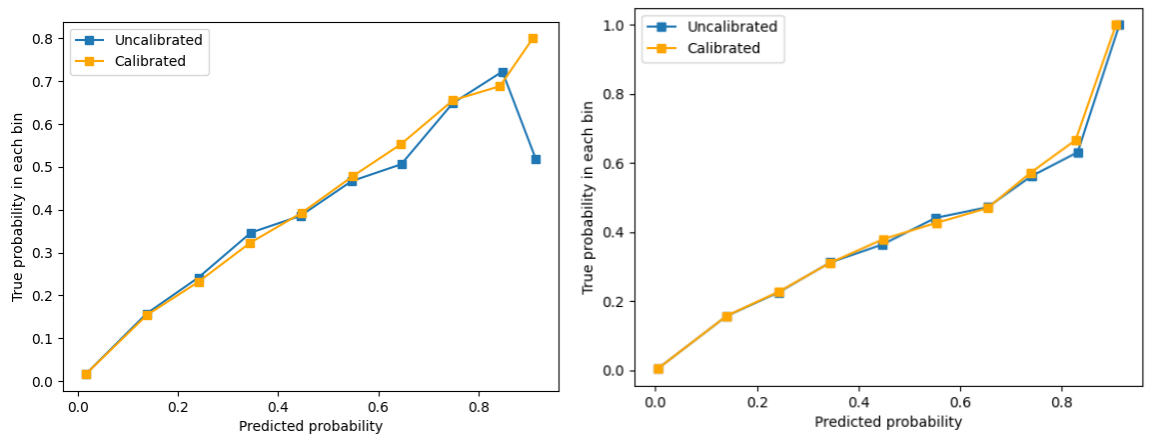
The calibration plots for the male and female models based on individual training data from 2002 to 2010. (A) Calibration plot of male model. (B) Calibration plot of female model. Participants were grouped in 10 bins based on the risk of suicide. “Uncalibrated” is the observed risk of suicide in each bin. “Calibrated” is the predicted risk of suicide in each bin.

The final models for males and females are in Tables 2 and 3, respectively. The model for males included 20 predictors, and the model for females had 22 predictors. The predictors in the models covered the levels of individual (eg, age and mental health physical diagnoses), health system (eg, hospitalization and mental health budget), and community (eg, material deprivation). The male and female models had common predictors (eg, age; living in rural area; hospitalization for suicide attempt; outpatient psychiatrist visits for mental health reasons; and presence of mood, anxiety, substance use, and personality disorders); some predictors are sex specific (in males: Charlson score, emergency and general physician [GP] visits for physical health reasons, and regional mental health budget; in females: material deprivation score and emergency and GP visits for mental health reasons). The sex-specific models had good discrimination (male model: C=0.79, 95% CI 0.78-0.79; female model: C=0.85, 95% CI 0.84-0.86) and calibration (Brier score male model 0.01; Brier score for female model 0.005). Figure 1 shows the visual comparison between the predicted and observed risk of suicide. The models calibrated well with the data, especially the model for females.

We converted the developed models into synthetic estimation models as described in the Methods section and directly applied the models in the development (from 2002 to 2010) and validation (from 2011 to 2019) data. We estimated the annual prevalence of suicide in Quebec from 2002 to 2019 and compared it with the observed risk in each year (Table S1 in Multimedia Appendix 1). During this period, the annual prevalence of suicide in males steadily decreased from 27 per 100,000 in 2002 to 20 per 100,000 in 2019, while the annual prevalence of suicide in females remained stable at around 6 per 100,000 to 7 per 100,000. The predicted annual prevalence of suicide in males and females based on the synthetic estimation models was very close to the observed proportions. Over 18 years, the synthetic estimation models had 1 per 100,000 over- or underestimation in 8 years for males and only in 3 years for females. For the rest of the years, the synthetic estimations were exactly the same as the observed proportions of suicide in the population. The good performance of the synthetic estimation models was also reflected by the small MAE and RMSE, with most of the RMSEs around 0.1. With the validation data from 2011 to 2019, the synthetic estimation model for males correctly predicted 4 of 5 high-risk regions in 8 years, and the model for females correctly predicted 4 of 5 high-risk regions in 5 years (Table 4).

To examine the accuracy and fairness of model prediction, we validated the synthetic estimation models by age groups and health regions using the 2019 data. As seen in Table S2 in Multimedia Appendix 1, the models performed well in different age groups. In 2019, the prevalence of suicide varied by health region, ranging from 14 per 100,000 (Montreal) to 275 per 100,000 (Nunavik) in males and from 4 per 100,000 (Laval) to 92 per 100,000 (Nunavik) in females. The models predicted the same regional variations in males and females with small absolute differences (Table S3 in Multimedia Appendix 1).

**Table 1:** The sociodemographic characteristics of the participants in the training data (2002-2010).

Variables		Men			Women	
		Control (n=316,574), n (%)	Suicide (n=6713), n (%)	Control (n=329,016), n (%)		Suicide (n=2186), n (%)
**Age (years)**						
	15-39	122,712 (38.76)	1948 (29.01)	120,116 (36.50)		612 (27.99)
	40-59	108,687 (34.33)	2998 (44.65)	108,740 (33.05)		1043 (47.71)
	≥60	85,175 (26.90)	1767 (26.32)	100,160 (30.44)		531 (24.29)
**Urbanicity^a^**						
	Rural town	62,682 (19.80)	1962 (29.22)	60,449 (18.37)		478 (21.86)
**Social deprivation score^b^**						
	1 (most privileged)	60,096 (18.98)	1167 (17.38)	59,127 (17.97)		310 (14.18)
	2	60,283 (19.04)	1323 (19.70)	60,002 (18.23)		339 (15.50)
	3	59,937 (18.93)	1223 (18.21)	61,491 (18.68)		358 (16.37)
	4	58,681 (18.53)	1162 (17.30)	62,807 (19.08)		427 (19.53)
	5 (most deprived)	58,620 (18.51)	1427 (21.25)	63,683 (19.35)		592 (27.08)
**Material deprivation score^b^**						
	1 (most privileged)	58,609 (18.51)	875 (13.03)	62,815 (19.09)		323(14.77)
	2	58,650 (18.52)	1034 (15.40)	61,194 (18.59)		365 (16.69)
	3	60,167 (19.00)	1274 (18.97)	61,428 (18.67)		378 (17.29)
	4	60,102 (18.98)	1450 (21.59)	61,349 (18.64)		449 (20.53)
	5 (most deprived)	60,089 (18.98)	1669 (24.86)	60,324 (18.33)		511 (23.37)

^a^Missing: men in control group: n=4611, 1.45%; men in suicide group: n=30, 0.44%; women in control group: n=3196, 0.97%; women in suicide group: n=6, 0.22%.

^b^Missing: men in control group: n=18,957, 5.98%; men in suicide group: n=411, 6.12%; women in control group: n=21,906, 6.65%; women in suicide group: n=160, 7.31%.

**Table 2:** The predictive model for suicide in males based on individual data from 2002 to 2010^a^.

Variables	Coefficient	Odds ratio	*P* value
Age	0.01	1.01	<.001
Charlson score	0.11	1.12	.01
Rural town _60m	0.44	1.55	<.001
Hospitalisation for Suicide attempt_60m	1.88	6.55	<.001
Emergency room visits for Physical health reasons_3m	1.00	2.72	<.001
Outpatient psychiatrist visits for mental health reasons_60m	0.67	1.95	<.001
Outpatient GP^b^ visits for Physical health reasons_60m	–0.15	0.86	<.001
Psychotherapy visits with a GP_3m	0.24	1.27	<.001
Mood and anxiety disorders_60m	0.99	2.69	<.001
Substance use disorders_60m	0.89	2.44	<.001
Personality disorders_60m	0.41	1.51	<.001
Respiratory disorders_60m	–0.28	0.76	<.001
Other mental disorders_60m	0.26	1.30	<.001
Symptoms, Signs and Ill-defined Conditions_3m	0.13	1.14	<.001
Non-intentional trauma _48m	0.21	1.23	<.001
Infectious disease_6m	0.30	1.35	<.001
Endocrine system disorder_48m	–0.15	0.86	<.001
Genito-urinary disorders_24m	–0.08	0.92	.01
Cancer_60m	–0.15	0.86	<.001
Regional mental budget	–0.001	1.00	<.001
Constant	–4.85	—^c^	—

^a^Receiver operating characteristic curve 0.79 (95% CI 0.78-0.79); Brier score 0.01.

^b^GP: general physician.

^c^Not applicable.

**Table 3:** The predictive model for suicide in females based on individual data from 2002 to 2010^a^.

Variables	Coefficient	Odds ratio	*P* value
Age	0.00	1.00	<.001
Rural town _48m	0.19	1.21	<.001
Material deprivation_2	–0.06	0.94	.38
Hospitalisation for Suicide attempt_60m	1.85	6.36	<.001
Hospitalisation for Physical health reasons_24m	0.35	1.42	<.001
Hospitalisation for mental health reasons_36m	0.37	1.45	<.001
Duration of hospitalisation for Physical health reasons_6m	0.007	1.01	.01
Emergency room visits for mental health reasons_60m	1.13	3.10	<.001
Outpatient psychiatrist visits for mental health reasons_60m	0.66	1.93	<.001
Outpatient GP^b^ visits for mental health reasons_36m	–0.01	0.99	.94
Mood and anxiety disorders_12m	1.05	2.86	<.001
Bipolar disorders_60m	0.08	1.08	.31
Substance use disorders_60m	0.84	2.32	<.001
Endocrine system disorder_60m	–0.25	0.78	<.001
Personality disorders_60m	0.47	1.60	<.001
Dementia_60m	–0.65	0.52	<.001
Genito-urinary disorders_48m	–0.23	0.79	<.001
Symptoms, Signs and Ill-defined Conditions_12m	0.22	1.25	<.001
Non-intentional trauma _36m	0.41	1.51	<.001
Respiratory disorders_24m	–0.10	0.90	.04
Other mental disorders_60m	0.10	1.11	.02
Infectious disease_36m	0.13	1.14	.01
Constant	–6.47	—^c^	—

^a^Receiver operating characteristic curve 0.85 (95% CI 0.84-0.86); Brier score 0.005.

^b^GP: general physician.

^c^Not applicable.

**Table 4:** The performance of the synthetic estimation models by years.

Years		Mean average error	Root mean square error	Proportion correctly predicted
**Model for males**				
	2011	0.03	0.13	0.6
	2012	0.03	0.14	0.8
	2013	0.03	0.13	0.8
	2014	0.03	0.13	0.8
	2015	0.03	0.13	0.8
	2016	0.03	0.17	1
	2017	0.02	0.17	0.8
	2018	0.03	0.17	0.8
	2019	0.02	0.16	0.8
**Model for females**				
	2011	0.009	0.09	0.6
	2012	0.009	0.09	0.6
	2013	0.009	0.09	0.6
	2014	0.01	0.10	0.8
	2015	0.01	0.10	0.8
	2016	0.01	0.1	1
	2017	0.01	0.1	0.6
	2018	0.01	0.1	0.8
	2019	0.009	0.09	0.8

## Discussion

### Principal Results

The data showed a sex difference in the trend of suicide risk and considerable variations in suicide risk by health regions in Quebec. The suicide risk in males had decreased since 2002, while the risk remained stable in females. This study demonstrated the feasibility of integrating individual-, program-, health care system–, and community-level data to build accurate prediction models for suicide at the population level. The models performed well in predicting suicide at both the provincial and health regional levels. The absolute difference between the observed and predicted proportions of suicide ranged from 0 to 1 per 100,000. The RMSEs were under 0.2. The prediction models could correctly identify the health regions that were at the top risk level, and the models achieved good performance in different age groups and health regions.

### Limitations

This study had several limitations. First, data about social determinants of health, medication use, the use of crisis hotlines, and access to lethal means were not available in the health administrative data. As such, we were not able to examine the extent to which these factors may improve the performance of the models. Second, although the predictors in the models were associated with suicide, causal inferences cannot be made. The goal of risk prediction models is to identify a key set of factors that in combination best predict the outcome. The models are not meant to test a hypothesis or make inferences about etiology. Third, the relationships between the selected factors and suicide are complex. The logistic regression model is a linear function. Although we found no evidence of interactions among the selected predictors, nonlinear relationships between some predictors and suicide are still possible. Future studies may test if models using machine learning techniques have a better performance.

### Comparison With Prior Works

The many factors in the models (eg, older age, living in rural areas, hospitalization for suicide attempt, emergency department visits for mental and physical health problems, and diagnosis of a mood/anxiety or substance use–related disorder) were associated with increased risk of suicide, which was consistent with the literature [6]. Outpatient GP visits for mental health (in females) and physical health (in males) were negatively associated with suicide in multivariate models. Audits of suicide cases, if aggregated, pointed toward the deficits in the health, mental health, and addiction services systems [8]. The work conducted in Quebec by our group [30] recommended better detection and treatment of substance use disorders at the primary (GPs) and specialist medical care levels, access to GPs and psychotherapy for common mental disorders and substance use disorders in the primary care context, mobile crisis teams operating from the emergency room (ER), public campaign targeting men about depression and substance use disorders as treatable diseases, and increasing the specialist mental health and addictions services budgets. This may explain the relationships between GP visits for mental health problems, patients with visits to their GP for endocrine or genitourinary system–related problems, dementia, and the decreased risk of suicide because these individuals have had the opportunity to be diagnosed with depression and be supported. Patients with infectious diseases secondary to comorbid substance use disorders may have been undetected and untreated, which may explain the negative association. Similarly, at the program level, deficits in the coordination of specialist addiction and mental health services for known patients seen at the ER were found in one-third of the cases, which may explain the negative association with ER visits. Finally, the increased mental health and addiction services was a key system-level recommendation by the Coroner’s Office to the provincial public-managed care system that can be implemented in the allocation of regional health budgets [31]. Although material deprivation was negatively associated with suicide in females, the association was not statistically significant. More granular data about socioeconomic status may provide more insights about the sex differences.

One unique feature that differentiates population risk prediction from individual risk prediction is the use of community-level characteristics as predictors. The data for estimating these community-level parameters may come from various sources. For example, Kandula et al [19] modeled county-level suicide risk in the United States using county-level predictors derived from 8 different sources (government programs, health surveys, and private organizations). For some predictors such as the prevalence of major depressive episodes, only state-level estimates were available and these estimates were extrapolated to the counties [19]. Hudson [32] explored the utility of a regression synthetic estimation model that incorporated individual data from the National Comorbidity Survey, census, and hospital administrative data to predict state-level prevalence of severe mental illness. The advantages of these population risk prediction models are the use of community-level predictors from existing sources or published research and the ability to adapt the models to the local context. Notably, our study used the regression synthetic estimation modeling approach. We used the provincial health administrative data and the CANUE indicators from a single source (ie, INSPQ). The use of a single data source may improve the efficiency of data analysis, data access, and eventually the decision-making process. On the other hand, the use of a single data source may miss some important information. In Canada, the provincial health administrative data are collected primarily for billing and administration purposes. It does not include granular data about social determinants of health (eg, race/ethnicity, poverty, employment, and housing quality), crime rate, social support, the use of crisis hotlines, access to lethal means, access to private health care, and medication use (except for children, youth, and seniors). These factors have been found to be associated with suicide risk, but pertinent data are collected and maintained by different organizations. Future studies should investigate how important data from different sources may be feasibly and efficiently integrated, which factors can improve the model performance, and the feasibility of local adaptation and implementation of the developed models.

One critical element of building risk prediction models is assessing model performance and model validation. This is to ensure that the developed model is accurate and has good performance in different populations or at different time periods. In this study, we developed the models using data from 2002 to 2010 and validated the models using the data from 2011 to 2019. Furthermore, we validated the models in different age groups and health regions. These models were designed to predict population risk and to identify high-risk regions/communities, not to be used by clinicians to identify high-risk individuals. Therefore, the focus of model performance assessment can be different. Specifically, the few existing population risk prediction models for suicide and mental illness focused on model calibration. Kandula et al [19] used symmetric proportional error (observed deaths – predicted deaths)/(observed deaths + predicted deaths) to quantify model calibration. Hudson [32] calculated the absolute difference between the predicted and observed prevalence of severe mental illness. In this study, we calculated the absolute difference between the observed and model-predicted proportions of suicide. Additionally, following the approach of Marks et al [33], we used the MAE, RMSE, and the proportion of correct identifications of high-risk regions as the indicators for model calibration. There is no consensus about the thresholds for absolute difference between predicted and observed proportions of correct identification. Consultations with knowledge users are needed to understand what indicators are informative about model performance and the level of the model error that is acceptable.

The results of this study are expected to have implications for population mental health planning. Few would deny that resource allocation should be partly driven by needs, and needs assessments typically require the knowledge of potential changes in prevalence estimates and in local population profiles (eg, their demographics, diagnoses, and mental health services use). The prediction models developed by this study will allow decision makers and mental health service planners to forecast the proportions of suicide in the years to come at the provincial (state) and health regional (county) levels based on the potential changes in local population profiles. Such profile changes may be estimated using health administrative data and national population census data. Additionally, region-specific risk estimates can help categorize health regions (eg, regions with relatively stable suicide risk, especially those that remained in the highest or lowest groups, or regions in which the largest year-to-year changes are observed) and hence help identify areas in greater need of preventive resources or, conversely, identify areas where interventions seem to be effective. Furthermore, if the data about the predictors are available on a more frequent basis (eg, monthly or biweekly), the models will support the development of nowcast suicide surveillance systems.

### Conclusions

Accurate prediction of the population suicide risk can play an important role in suicide prevention. This information can allow policy and decision makers and mental health service planners to categorize regions/communities that are at high risk and to monitor changes in risk so that they may mobilize resources [31] and interventions to the right populations and the right places at the right time. Using linked health administrative databases, our study demonstrated the feasibility and validity of developing prediction models for population suicide risk, incorporating individual-, health system–, and community-level variables. Routinely collected health administrative data are readily accessible to policy and decision makers and mental health service planners. Suicide risk prediction models based on health administrative data can provide useful information to policy/decision makers at the moment they need the information. This effort can be enhanced by timely access to other critical information at the population level. However, the methodology of population risk prediction should be further studied to enhance the validity and precision of population risk prediction.

## Appendix

Multimedia Appendix 1 Supplementary tables.

## Abbreviations

CANUE: Canadian Urban Environmental Health Research
ER: emergency room
GP: general physician
INSPQ: Quebec Institute of Public Health
LASSO: least absolute shrinkage and selection operator
MAE: mean average error
RMSE: root mean square error
