# Supplementary material for: Predicting the Population Risk of Suicide Using Routinely Collected Health Administrative Data in Quebec, Canada: Model-Based Synthetic Estimation Study
Source: JMIR Public Health Surveill. 2024 Jun 28;10:e52773. doi: 10.2196/52773 (PMC11245657; doi:10.2196/52773)
Supplement: Multimedia Appendix 1 [file publichealth_v10i1e52773_app1.docx]

**Supplement file:**

**Table S1: The predicted (synthetic) and observed risks of suicide from 2002 to 2019 in males and females in the province of Quebec from 2002 to 2019.**

| **Years** | **Observed risk n/100,000** | **Predicted (synthetic) risk n/100,000** | **Absolute difference n/100,000** |
| --- | --- | --- | --- |
| **Model for males** | | | |
| 2002 | 27 | 28 | -1 |
| 2003 | 25 | 25 | 0 |
| 2004 | 21 | 22 | -1 |
| 2005 | 24 | 24 | 0 |
| 2006 | 22 | 23 | -1 |
| 2007 | 22 | 22 | 0 |
| 2008 | 21 | 21 | 0 |
| 2009 | 21 | 20 | 1 |
| 2010 | 20 | 20 | 0 |
| 2011 | 21 | 22 | -1 |
| 2012 | 22 | 21 | 1 |
| 2013 | 22 | 21 | 1 |
| 2014 | 21 | 21 | 0 |
| 2015 | 21 | 21 | 0 |
| 2016 | 21 | 21 | 0 |
| 2017 | 21 | 21 | 0 |
| 2018 | 22 | 21 | 1 |
| 2019 | 20 | 20 | 0 |
| **Mode for females** | | | |
| 2002 | 7 | 7 | 0 |
| 2003 | 7 | 8 | -1 |
| 2004 | 7 | 7 | 0 |
| 2005 | 7 | 7 | 0 |
| 2006 | 6 | 7 | -1 |
| 2007 | 6 | 6 | 0 |
| 2008 | 7 | 7 | 0 |
| 2009 | 6 | 6 | 0 |
| 2010 | 6 | 6 | 0 |
| 2011 | 6 | 6 | 0 |
| 2012 | 6 | 6 | 0 |
| 2013 | 6 | 7 | -1 |
| 2014 | 7 | 7 | 0 |
| 2015 | 7 | 7 | 0 |
| 2016 | 7 | 7 | 0 |
| 2017 | 7 | 7 | 0 |
| 2018 | 7 | 7 | 0 |
| 2019 | 6 | 6 | 0 |

**Table S2. The predicted (synthetic) and observed proportions of suicide by age groups in 2019.**

| **Age groups** | **Observed risk n/100,000** | **Predicted risk n/100,000** | **Absolute difference n/100,000** |
| --- | --- | --- | --- |
| **Model for males** | | | |
| 15-39 | 16 | 16 | 0 |
| 40-59 | 21 | 22 | - 1 |
| 60+ | 21 | 21 | 0 |
| **Model for females** | | | |
| 15-39 | 5 | 5 | 0 |
| 40-59 | 10 | 8 | 2 |
| 60+ | 5 | 6 | -1 |

**Table S3: The predicted (synthetic) and observed proportions of suicide by health regions in 2019.**

| **Regions** | | **Observed risk n/100,000** | **Predicted risk n/100,000** | | **Absolute difference n/100,000** |
| --- | --- | --- | --- | --- | --- |
| **Model for males** | | | | | |
| Bas-Saint-Laurent | | 26 | 26 | | 0 |
| Saguenay - Lac-Saint-Jean | | 23 | 23 | | 0 |
| Capitale Nationale | | 22 | 22 | | 0 |
| Mauricie et Centre-du-Quebec | | 26 | 25 | | 1 |
| Estrie | | 25 | 25 | | 0 |
| Montreal | | 14 | 14 | | 0 |
| Outaouais | | 25 | 25 | | 0 |
| Abitibi-Temiscamingue | | 34 | 34 | | 0 |
| Cote-Nord | | 28 | 29 | | -1 |
| Nord-du-Quebec | | 31 | 29 | | 2 |
| Gaspesie - Iles-de-la-madeleine | | 34 | 35 | | -1 |
| Chaudiere-Appalaches | | 27 | 27 | | 0 |
| Laval | | 15 | 15 | | 0 |
| Lanaudiere | | 24 | 23 | | 1 |
| Laurentides | | 24 | 24 | | 0 |
| Monteregie | | 18 | 18 | | 0 |
| Nunavik | | 275 | 279 | | -4 |
| Terres-Cries-de-la-Baie-James | | 37 | 37 | | 0 |
| **Model for females** | | | | | |
| Bas-Saint-Laurent | 5 | | 6 | -1 | |
| Saguenay - Lac-Saint-Jean | 7 | | 8 | -1 | |
| Capitale Nationale | 8 | | 8 | 0 | |
| Mauricie et Centre-du-Quebec | 9 | | 9 | 0 | |
| Estrie | 7 | | 7 | 0 | |
| Montreal | 6 | | 6 | 0 | |
| Outaouais | 6 | | 6 | 0 | |
| Abitibi-Temiscamingue | 12 | | 11 | 1 | |
| Cote-Nord | 9 | | 10 | -1 | |
| Nord-du-Quebec | Not reportable | | Not reportable | Not reportable | |
| Gaspesie - Iles-de-la-madeleine | 7 | | 7 | 0 | |
| Chaudiere-Appalaches | 9 | | 9 | 0 | |
| Laval | 4 | | 4 | 0 | |
| Lanaudiere | 5 | | 5 | 0 | |
| Laurentides | 7 | | 7 | 0 | |
| Monteregie | 6 | | 6 | 0 | |
| Nunavik | 92 | | 84 | 8 | |
| Terres-Cries-de-la-Baie-James | Not reportable | | Not reportable | Not reportable | |

Not reportable: The number of suicide cases are too small or zero.
